# Nitrogen deprivation induces triacylglycerol accumulation, drug tolerance and hypervirulence in mycobacteria

**DOI:** 10.1038/s41598-019-45164-5

**Published:** 2019-06-17

**Authors:** Pierre Santucci, Matt D. Johansen, Vanessa Point, Isabelle Poncin, Albertus Viljoen, Jean-François Cavalier, Laurent Kremer, Stéphane Canaan

**Affiliations:** 1 Aix-Marseille Univ, CNRS, LISM, IMM FR3479, Marseille, France; 2 Institut de Recherche en Infectiologie de Montpellier (IRIM), CNRS, UMR 9004, Université de Montpellier, 34293 Montpellier, France; 3 INSERM, IRIM, 34293 Montpellier, France

**Keywords:** Pathogens, Lipidomics

## Abstract

Mycobacteria share with other actinomycetes the ability to produce large quantities of triacylglycerol (TAG), which accumulate as intracytoplasmic lipid inclusions (ILI) also known as lipid droplets (LD). *Mycobacterium tuberculosis* (*M. tb*), the etiologic agent of tuberculosis, acquires fatty acids from the human host which are utilized to synthesize TAG, subsequently stored in the form of ILI to meet the carbon and nutrient requirements of the bacterium during long periods of persistence. However, environmental factors governing mycobacterial ILI formation and degradation remain poorly understood. Herein, we demonstrated that in the absence of host cells, carbon excess and nitrogen starvation promote TAG accumulation in the form of ILI in *M. smegmatis* and *M. abscessus*, used as surrogate species of *M. tb*. Based on these findings, we developed a simple and reversible *in vitro* model to regulate ILI biosynthesis and hydrolysis in mycobacteria. We also showed that TAG formation is *tgs1* dependent and that lipolytic enzymes mediate TAG breakdown. Moreover, we confirmed that the nitrogen-deprived and ILI-rich phenotype was associated with an increased tolerance towards several drugs used for treating mycobacterial infections. Importantly, we showed that the presence of ILI substantially enhanced the bacterial burden and granuloma abundance in zebrafish embryos infected with lipid-rich *M. abscessus* as compared to embryos infected with lipid-poor *M. abscessus*, suggesting that ILI are actively contributing to mycobacterial virulence and pathogenesis.

## Introduction

Upon infection with *Mycobacterium tuberculosis* (*M. tb*), the causative agent of TB, less than 5% of infected people will progress towards active infection, whereas 95% remain classified as latently infected without any symptoms or signs of illness^1^. In these asymptomatic individuals, bacteria persist within granulomatous lesions in a non-replicating or “dormant” state, from which they may reactivate to induce active TB. It is hypothesized that these persistent bacterial sub-populations are characterized by both low metabolic activity and the presence of large lipid droplets (LD), referred to as intracytoplasmic lipid inclusions (ILI) that are filled with triacylglycerol (TAG)^2,3^. Interestingly, TAG synthesis is a conserved feature of several bacterial species belonging to the *Actinobacteria* phylum, consisting largely of soil dwelling bacteria and including the *Mycobacterium* genus^4–6^. The accumulated TAG constitute a major source of carbon and energy, sustaining survival of intra- and extracellular mycobacteria, and is also directly linked to division arrest, loss of acid-fastness and to increased drug tolerance^7–11^. In recent years, a large number of *in vitro* and *ex vivo* models have been developed to mimic the ILI-inducing environment encountered by mycobacteria within their hosts^12^. It has been demonstrated that within differentiated foamy macrophages (FM), the TAG content of lipid bodies (LB) can be hydrolysed and processed by intraphagosomal mycobacteria, thus leading to the formation of ILI^2,7,8,13^. Moreover, several studies have reported that ILI can also be synthesized by extracellular bacteria during stressful conditions and may be considered as a metabolic strategy employed by prokaryotic cells to survive under harsh environments^4,5,14,15^. While mycobacterial ILI formation has been observed in slow- and fast-growing mycobacteria such as species from the *M. tb* complex^9,16–21^, *M. leprae*^22^, *M. kansasii*^23^, *M. marinum*^24,25^, *M. avium*^8^, *M. abscessus*^26^ as well as saprophytic species such as *M. smegmatis*^9,27,28^, the role of ILI formation in host-pathogen interactions remains poorly understood.

To date, only a limited number of external stimuli promoting TAG synthesis have been identified. Among them, hypoxia was found to result in a non-replicating state and in ILI formation in *M. tb*^29,30^. Growth of slow-growing mycobacteria under hypoxic conditions, as described in the Wayne and Hayes dormancy model, triggers upregulation of the major TAG synthase, *tgs1*, in a DosR regulon dependent-manner^29–32^. These early observations led to the development of more complex systems, such as an *in vitro* multiple stress model combining hypoxia, low pH and exposure to nitric oxide, inducing TAG production and accumulation, loss of acid-fastness and tolerance to drugs^17^. Other studies, evoked by the fact that fatty acids are among the most abundant molecules *in vivo*, scrutinized the effect of long-chain fatty acid-supplemented medium on lipid metabolism^9,14,27,33^ and reported a similar transcriptional response to that observed in growing under hypoxia or within foamy macrophages. These findings emphasize a common transcriptional signature governing TAG synthesis in mycobacteria exposed to various stresses^14^. For *M. tb*, the use of host-derived lipids for ILI formation has been implicated as a process that provides bacilli with a carbon-based energy source which acts to limit metabolic stress and promote mycobacterial dormancy, ultimately supporting persistence within the host during lengthy periods of latent infection^2,7,13,34,35^. However, the biological factors governing TAG accumulation in the form of ILI and the contribution of these intracellular structures in mycobacterial survival and virulence during host-pathogen interaction remains elusive.

To better characterize the environmental conditions modulating TAG formation/consumption processes, the non-pathogenic strain *M. smegmatis* was initially used to demonstrate that nitrogen and carbon availability are two key metabolic factors governing TAG formation and accumulation in the form of ILI in mycobacteria. Importantly, these physiological processes are highly conserved in the opportunistic pathogen *M. abscessus*, a fast-growing non-tuberculous mycobacterium responsible for chronic pseudo-tubercular infection^36^. Following genetic, biochemical and imaging approaches, we have developed and validated a simple *in vitro* experimental procedure that allows *in vitro* regulation of ILI formation/degradation processes in mycobacteria. Finally, by using the well-established *M. abscessus*/zebrafish infection model^37,38^, we have demonstrated that ILI formation confers significant advantages to the bacteria in the establishment and progress of the infection *in vivo*.

## Results

### Excess of glycerol promotes TAG synthesis during stationary phase and this phenomenon is regulated by nitrogen availability

**Figure 1:**
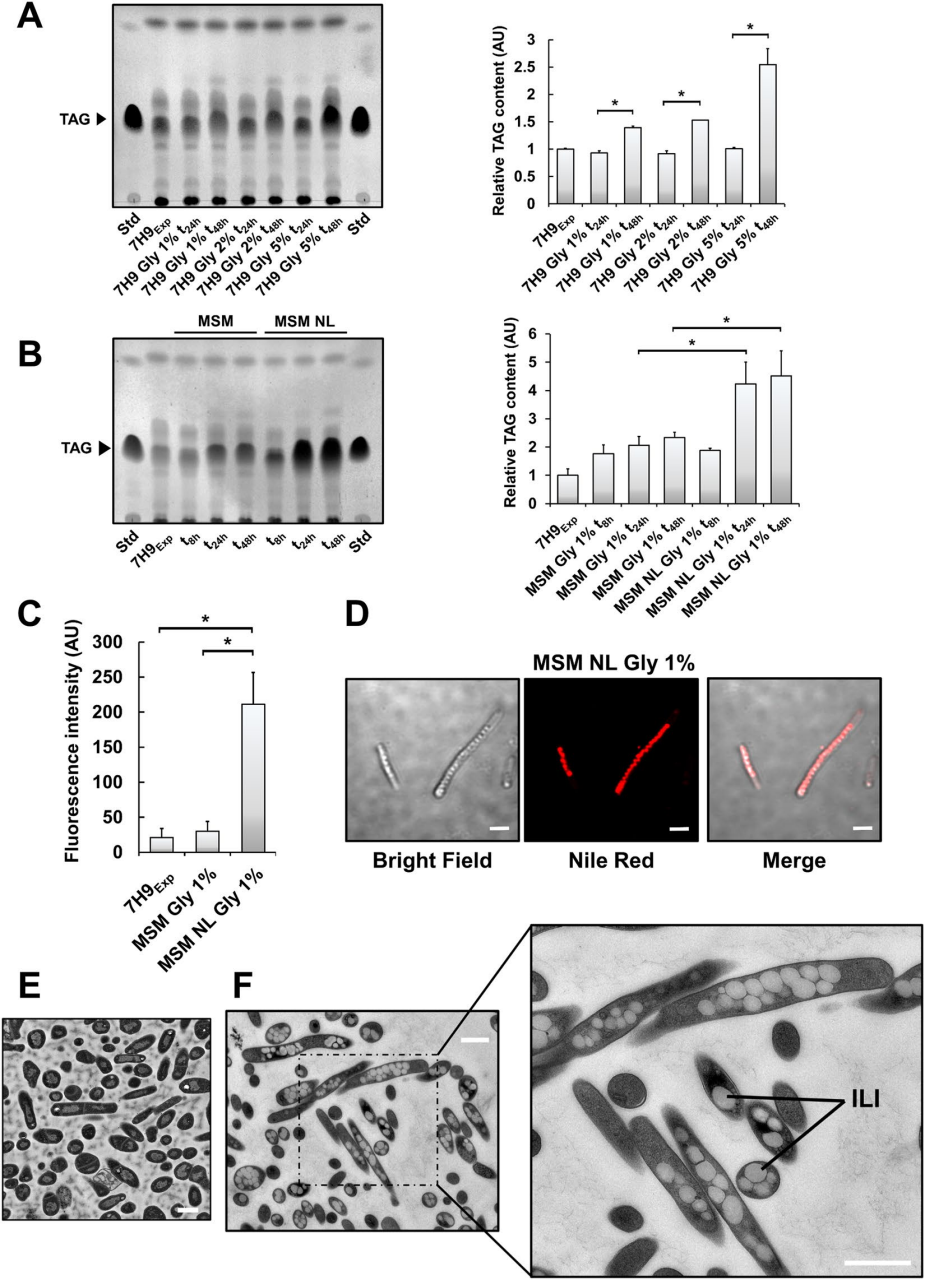
TAG accumulation under the form of ILI is driven by carbon excess and nitrogen starvation in stationary phase. (**A**) Mycobacterial cultures were grown in different media containing increasing glycerol concentrations (1%, 2% and 5%) and were collected at 24 h or 48 h, corresponding to exponential or stationary phase, lyophilized and finally the same amount of dry cell weight was used for apolar lipid extraction. Left panel corresponds to TLC plate analysis of TAG extracted from exponential and stationary-phase cultures with increasing concentrations of Gly, with triolein as standard. Right panel corresponds to TLC densitometric analysis of the relative TAG level in each sample with cultures in classic 7H9 (7H9_Exp_) used as reference. **(B)** Cultures grown in minimal salt medium containing either 1 g/L or 0.05 g/L of NH_4_Cl and 1% Gly as carbon source were collected after 8 h, 24 h or 48 h incubation periods, lyophilized and equal weights of dry cells used for apolar lipid extraction. TAG levels from each culture were analysed by TLC with triolein as standard. The TLC plate (left panel) is representative of two individual experiments. TLC densitometric analysis of relative TAG levels in each sample with cultures in exponential phase in classic 7H9 (7H9_Exp_) used as reference (right panel). All results are expressed as mean values ± SD of two independent experiments. TAG band intensities were compared using a one-way ANOVA test where * corresponds to a *p*-value < 0.05. **(C)** Average Nile-Red fluorescence intensity determined for 6 different 126 µm^2^ fields containing between 50 and 150 cells each. Fluorescence intensities were compared using a two-tailed Mann-Whitney test where * corresponds to a *p*-value < 0.05. **(D)** Phase contrast, Fluorescence and Merge channels of *M. smegmatis* cells grown for 24 h in MSM NL Gly 1% medium. Cells harbour distinct morphologies and contain ILI occupying most of the cytoplasm space. Scale bars represent 2 µm. Cells were fixed with glutaraldehyde and processed for EM. **(E)** Thin section of an *in vitro* culture of *M. smegmatis* in classical 7H9 medium. The scale bar represents 1 µm. **(F)** Thin section of an *in vitro* culture of *M. smegmatis* in MSM NL Gly medium. Right panel is a zoomed-in picture providing a better view and resolution of ILI. Scale bars represent 2 µm.

To examine the role of carbon and nitrogen availability in TAG accumulation, the non-pathogenic strain *M. smegmatis* mc^2^155 was grown in Middlebrook 7H9 broth supplemented with increasing glycerol (Gly) concentrations. Apolar lipids extraction (comprising TAG) during exponential growth (24 h) or stationary phase (48 h) was performed prior to thin layer chromatography (TLC) analysis. Cultures containing higher Gly concentrations at 48 h exhibited increased TAG levels by 1.5-fold (*i.e*., 1% and 2% Gly) to 2.5-fold (*i.e*., 5% Gly) as compared to standard 7H9 medium (7H9_Exp_) (Fig. 1A and Fig. S1).

To investigate the effect of nitrogen requirements for TAG biosynthesis, *M. smegmatis* was grown in well-defined Minimal Mineral Salt Medium supplemented with either high nitrogen (MSM; containing 1 g/L NH_4_^+^) or low nitrogen (MSM NL; containing 0.05 g/L NH_4_^+^) concentrations, and 1% Gly as the sole carbon source. Cells were grown in either MSM Gly or MSM NL Gly media and their respective growth curves determined (Fig. S2A). Typically, *M. smegmatis* culture reached maximal OD_600nm_ values of around 3.9 in MSM Gly medium after 24–30 h of incubation, while in MSM NL Gly the OD_600nm_ values reached a plateau 1.8–1.9. No bacterial growth was observed in nitrogen-limiting minimal medium (MSM N^−^) containing 1% Gly. These observations emphasize the crucial role of nitrogen availability for mycobacterial division and biomass production.

To better define the impact of nitrogen limiting conditions on TAG formation, *M. smegmatis* was grown in either MSM Gly 1% or MSM NL Gly 1% prior to apolar lipid extraction and TAG quantification at various time points (Fig. 1B). In MSM Gly 1% at 48 h, *M. smegmatis* harboured 2.3 times greater quantities of TAG compared to the control sample grown in 7H9_Exp_. This phenomenon was more pronounced after 24 h and 48 h of growth in MSM NL Gly 1% medium, with relative TAG levels representing a similar fold-change of 4.2 ± 0.8 and 4.5 ± 0.9, respectively, as compared to standard growth conditions (7H9_Exp_) and nearly double that in MSM Gly 1% medium. These results clearly show that nitrogen starvation strongly stimulates TAG formation in *M. smegmatis*.

### Nitrogen and carbon availability regulate TAG biosynthesis and storage under the form of ILI

To further confirm that nitrogen starvation triggers accumulation of newly produced TAG in the form of ILI, *M. smegmatis* were labelled at 24 h with Nile Red, a fluorescent dye that primarily stains neutral lipids and phospholipids (Fig. 1C,D). The fluorescent signal from cells grown in either 7H9_Exp_ or MSM Gly 1% medium was low and mainly peripheral, suggesting that the cell wall-associated lipids were stained (data not shown). Conversely, bacteria in MSM NL Gly1% harboured a brighter, more intense and compact cytoplasmic signal, presumably due to ILI staining (Fig. 1D). Quantitative analysis of the fluorescent signal showed that bacteria in 7H9_Exp_ and in MSM Gly 1% emitted 21 ± 13 and 30 ± 15 fluorescence units, respectively (Fig. 1C). As expected, bacteria exposed to MSM NL Gly 1% were approximately 7 to 10 times more fluorescent, with a mean value of 211 ± 45 fluorescence arbitrary units (Fig. 1C). By comparing the relative fluorescence intensity ratios between each culture condition, our data is seemingly in agreement with previously published quantification of lipid-rich *vs* lipid-poor mycobacteria^28^, further highlighting the role of nitrogen and carbon availability in regulating TAG biosynthesis.

To gain insight into the nature and features of these lipid inclusions, *M. smegmatis* grown in MSM NL Gly 1% for 24 h was fixed with glutaraldehyde and processed for transmission electron microscopy (TEM). Bacteria in 7H9_Exp_ used as control sample were devoid of ILI, as anticipated (Fig. 1E). In sharp contrast, when grown in MSM NL Gly 1%, the bacilli displayed multiple and large ILI. While more than 90% of the population analysed on grids were ILI-positive, the number and size of ILI differed strongly between individual bacterial cells (Fig. 1E,F), suggesting that phenotypic heterogeneity occurs under these growth conditions. The relative abundance of each type of ILI profile (Materials and Methods, Fig. S3) showed that nearly 27 ± 11% and 57 ± 8% of the bacterial population belong to the large ILI^2+^ and ILI^3+^ categories^8,26^, respectively, illustrating the very high TAG content in these bacteria.

### Nitrogen-deprived and lipid-rich M. smegmatis are phenotypically tolerant to frontline anti-tubercular drugs

It has previously been shown in slow-growing and rapid-growing mycobacterial species that phenotypic antibiotic resistance is closely associated with TAG accumulation^11,17^. This prompted us to explore the drug susceptibility profile of *M. smegmatis* to three first-line antibiotics in 7H9_Exp_, MSN Gly 1% and MSM NL Gly 1% (Table 1). The concentration of isoniazid (INH) or rifampicin (RIF) leading to 90% bacterial growth inhibition (MIC_90_) was nearly 10-fold higher when *M. smegmatis* was grown in MSM NL Gly 1% as compared to growth in 7H9_exp_, while intermediate values were obtained when grown in MSM Gly 1%. These results are in agreement and correlate with the levels of TAG produced under the similar growth conditions. However, the ILI profile failed to correlate with tolerance to kanamycin (KAN), a drug able to act both on replicating and dormant/persistent bacteria^39^. The percentage of *M. smegmatis* that became tolerant to RIF and INH was determined after 24 h of exposure to antibiotics using the agar plating method. Tolerance to RIF and INH of bacteria grown in MSM NL Gly 1% was about 2.9-fold (62.6% survival) and 2.5-fold (100% survival) higher than that of bacteria grown in 7H9_exp_ (Table 1).

**Table 1:** Drug susceptibility testing of lipid-poor (7H9_Exp_) and lipid-rich (MSM NL Gly 1% medium) *M. smegmatis*. Evaluation of antibiotic tolerance was performed using the agar plating method.

*Culture conditions*	MIC_90_ (µg/mL)^a^	Percentage of the *M. smegmatis* tolerant population to antibiotics^b^				
	RIF	INH	KAN	RIF (12 µg/mL)	INH (12 µg/mL)	KAN (10 µg/mL)
7H9_Exp_ – 24 h	2.6	3.1	0.94	22 ± 2	41 ± 4	0 ± 0
7H9_Exp_ – 48 h.	2.6	3.1	0.94	—	—	—
MSM Gly 1% – 24 h.	13.8	>30	0.73	43 ± 5	80 ± 9	0 ± 0
MSM Gly 1% – 48 h.	12.7	>30	0.7	—	—	
MSM NL Gly 1% – 24 h	>30	>30	0.84	62 ± 1.0	100 ± 6	0 ± 0
MSM NL Gly 1% – 48 h	>30	>30	0.88	—	—	—

^a^MIC_90_: compound minimal concentration leading to 90% bacterial growth inhibition determined based on the REMA assay. Values are mean of two independent assays performed in duplicate (CV% < 5%). ^b^*M. smegmatis* cells were cultured in their respective medium at 37 °C for 24 h and were further treated with each antibiotic for additional 24 h. The viable cells were quantified using the agar plating method. Untreated cells were used as control representing 100% of cell viability.

### Carbon/nitrogen ratio governs ILI formation in M. abscessus

**Figure 2:**
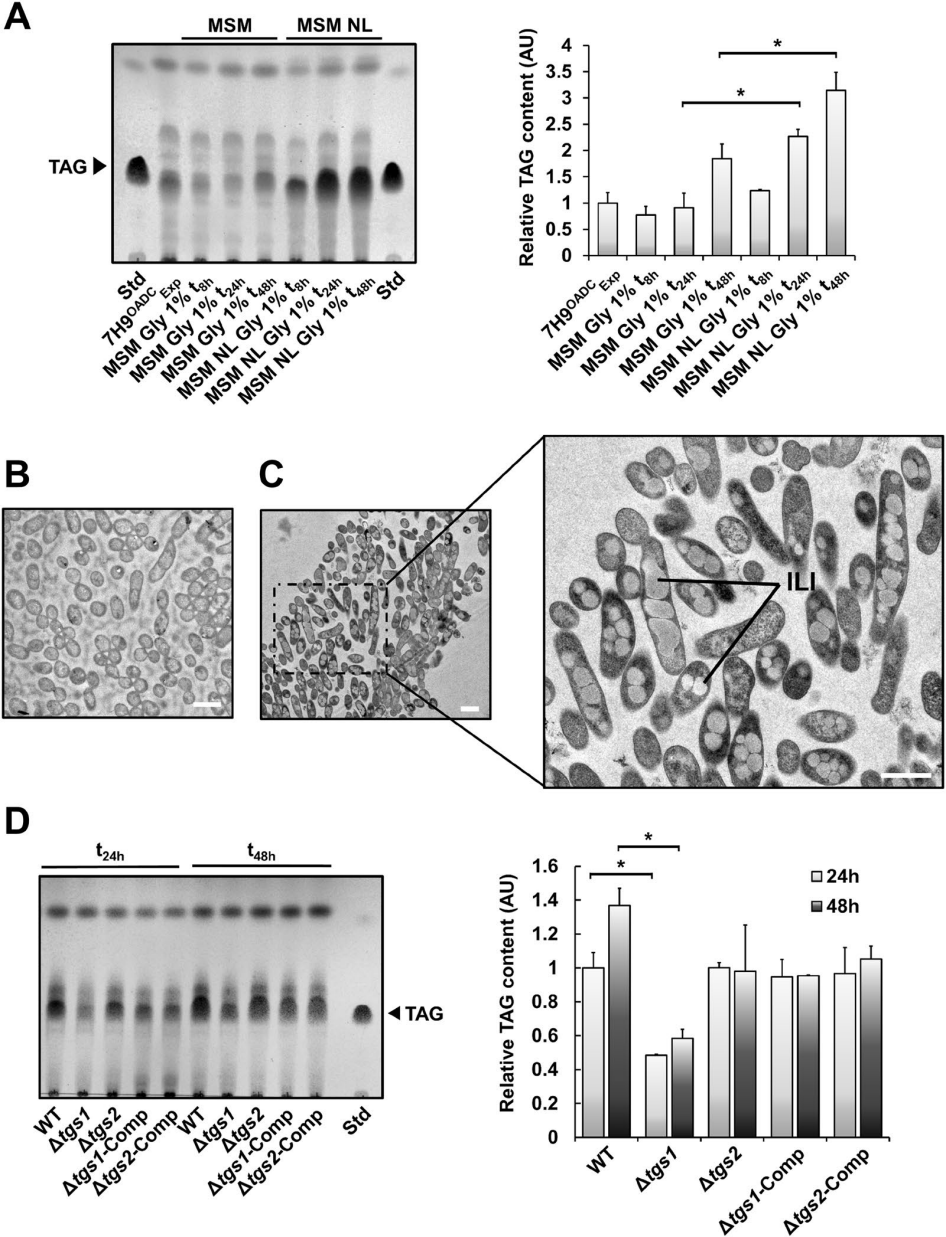
Nitrogen/carbon ratio governs TAG accumulation as ILI in *Mycobacterium abscessus*. (**A**) *M. abscessus* cells were grown in minimal salt medium containing either 1 g/L or 0.05 g/L NH_4_Cl and 1% Gly as sole carbon source. Cultures were collected after an 8 h, 24 h or 48 h incubation period, lyophilized and equal amounts of dry cells used for apolar lipid extraction. TAG levels from each culture were analysed by TLC with triolein as standard. The TLC plate (left panel) is representative of two independent experiments. Right panel corresponds to TLC densitometric analysis of relative TAG levels in each sample, with cultures in exponential phase in classic 7H9TG^OADC^ used as reference. Results are expressed as mean values ± SD of two individual experiments. TAG band intensities were compared using a one-way ANOVA test where * corresponds to a *p*-value < 0.05. **(B)** Thin section of an *in vitro* culture of *M. abscessus* in classical 7H9^OADC^ medium. The scale bar represents 1 µm. **(C)** Thin section of an *in vitro* culture of *M. abscessus* in MSM NL Gly 1% medium. Right panel is a zoomed-in picture providing a better view and resolution of ILI. Scale bars represent 1 µm. **(D)** TAG levels from *M. abscessus* grown in MSM NL Gly. Cultures were collected after a 24 h or 48 h incubation period, lyophilized and equal weights of dry cells used for apolar lipid extraction. TAG levels from WT, Δ*tgs1*, Δ*tgs2* and their respective complemented strains were analysed by TLC with triolein as standard. TLC densitometric analysis of relative TAG levels in each genetic background, with cultures from WT *M. abscessus* used as a reference. Results are expressed as mean values ± SD of two independent experiments. TAG band intensities of WT and Δ*tgs1* were compared using a one-way ANOVA test where * corresponds to a *p*-value < 0.05.

To define whether the stress response to nitrogen deprivation is conserved among other mycobacterial species, *M. abscessus*; an opportunistic pathogenic agent responsible for long-term persistent infection^36^ and previously demonstrated to accumulate ILI within FMs^26^ was used. The *M. abscessus* type strain CIP104536^T^ was grown in MSM Gly 1% or in MSM NL Gly 1% (Fig. S2B,C) with TAG content monitored over time (Fig. 2A). TLC analysis revealed that upon nitrogen starvation, *M. abscessus* smooth morphotype (S) produces 2.5 and 1.7 times more TAG in MSM NL Gly 1% medium as compared to MSM Gly 1% after 24 and 48 h of incubation (Fig. S4A,B), respectively, similar to prior observation in *M. smegmatis* (Fig. 1B). Following TEM analysis, we found that, in contrast to the control sample in standard 7H9^OADC^ broth (Fig. 2B), ILI were clearly visible in bacteria grown in MSM NL Gly 1% (Fig. 2C). Quantitative analyses revealed that 36 ± 4% of *M. abscessus* displayed an ILI^3+^ profile, approximately two-fold less than the proportion of ILI^3+^ profiles observed for *M. smegmatis* grown under the same conditions, highlighting a species-specific difference (Fig. S3).

### Tgs1 operates under nitrogen-deprived conditions to produce TAG

It has been recently demonstrated that ILI formation within FMs is dependent on *tgs1*, the major TAG synthase-encoding gene in *M. tb* and *M. abscessus*^7,26^. To address whether TAG accumulation during nitrogen starvation requires the same factors both *in vitro* and *ex vivo*, we used the genetically defined Δ*tgs1* and Δ*tgs2* mutants, in which *tgs1* and *tgs2* have been disrupted in *M. abscessus* S along with their respective complemented strains^26^. Each strain was grown in MSM NL Gly 1% for 24 h and 48 h, harvested and analyzed by TLC for their lipid content (Fig. 2D). Quantitative densitometric analysis showed that the TAG production was impaired by 52 and 62% in Δ*tgs1* after 24 and 48 h of incubation in MSM NL Gly 1%, respectively, as compared to the parental strain (Fig. 2D). Functional complementation, achieved by introducing pMV261*::tgs1* promoting a strong and constitutive production of Tgs1, resulted in almost complete TAG synthesis restoration (Fig. 2D). In line with previously reported results^26^, these findings confirm the major contribution of Tgs1, but not of Tgs2, in ILI formation in *M. abscessus*.

### ILI formation and hydrolysis is a reversible phenomenon mediated by lipolytic enzymes

**Figure 3:**
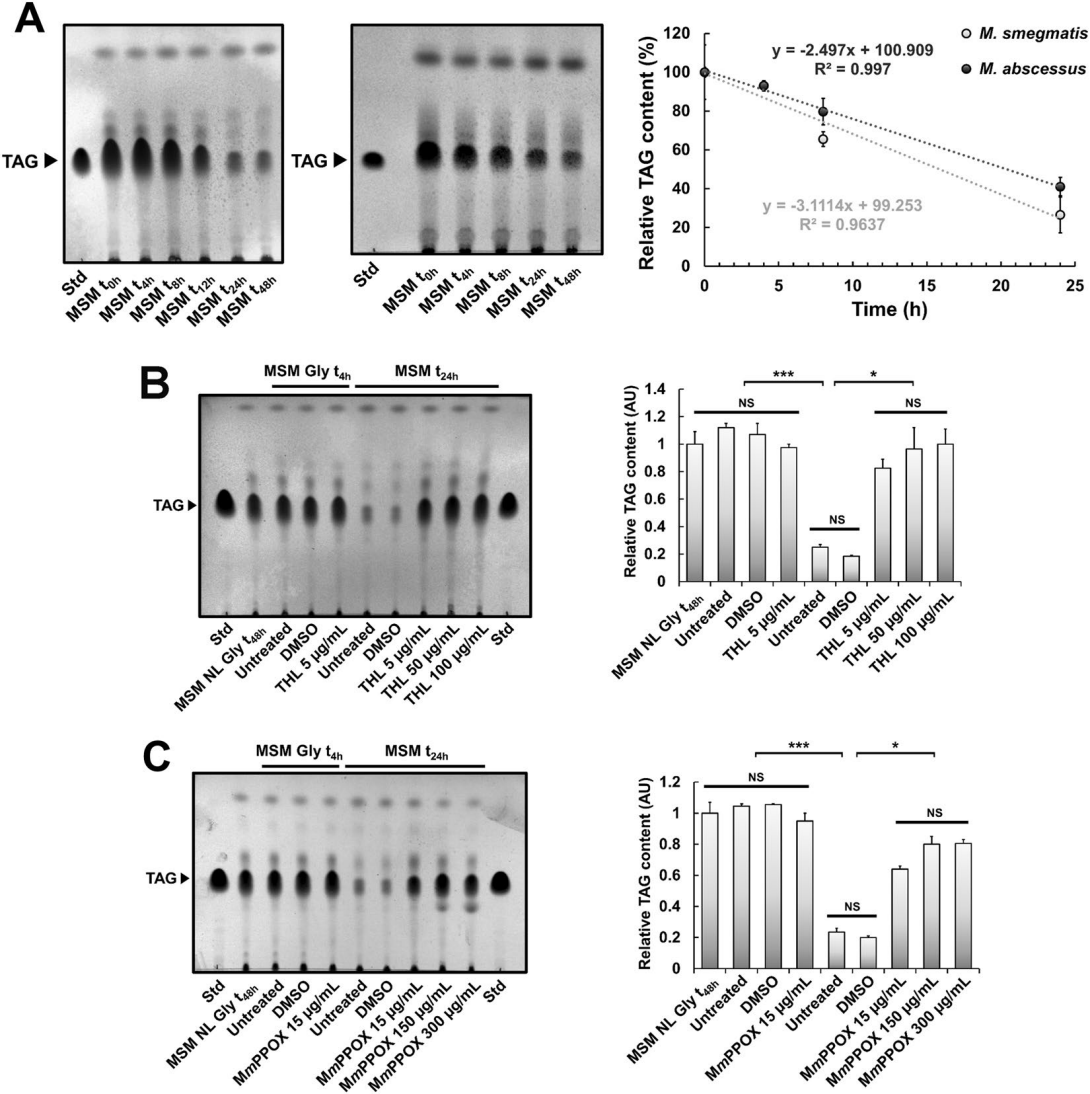
ILI hydrolysis is a reversible and rapid phenomenon upon carbon starvation and is mediated by lipolytic enzymes. **(A)** ILI hydrolysis occurs rapidly during carbon starvation. Lipid rich mycobacterial cultures were transferred into a carbon starved-MSM medium and collected at indicated time points, lyophilized and equal amounts of dry cells used for apolar lipid extraction. TAG levels from *M. smegmatis* (left panel) and *M. abscessus* (right panel) were analysed by TLC with triolein as standard. Each TLC plate is representative of individual experiments performed in triplicate. Variation of relative TAG content, assessed by densitometric analysis of the TLC plates obtained from *M. smegmatis* and *M. abscessus* cultures, as a function of time. Results are expressed as mean values ± SD of two independent experiments. (**B-C**) TAG hydrolysis and ILI consumption can be pharmacologically blocked by serine-hydrolase inhibitors. Lipid rich mycobacterial cultures were harvested, re-suspended in MSM Gly, pre-incubated with or without THL **(B)** or M*m*PPOX **(C)** for 4 h, centrifuged and finally incubated in MSM medium devoid of carbon for 24 h with the indicated concentration of THL (stock solution 5 mg/mL in DMSO) or M*m*PPOX (stock solution at 5 mg/mL in DMSO). Cells were collected at specific time points and their lipids were extracted and analysed by TLC analysis. Each TLC plate is representative of two independent experiments. TAG levels from each *M. smegmatis* culture were analysed by TLC with triolein as standard. Results from densitometry are expressed as mean values ± SD of two independent experiments. TAG band intensities of lipid rich, lipid poor (devoid of inhibitors) and lipid-rich inhibited cells (THL or M*m*PPOX) were compared using a one-way ANOVA test where * corresponds to a *p*-value < 0.05 and *** to a *p*-value < 0.001.

It has been emphasized that ILI breakdown occurring upon carbon-limited conditions in *M. tb* could mimic reactivation from the latency phase^19,28^. To monitor TAG consumption in *M. smegmatis* (Fig. 3A, left panel) and *M. abscessus* (Fig. 3A, right panel), both species were grown in MSM NL Gly 1% for 48 h until high levels of TAG were reached. Pellets were then washed and re-suspended in fresh MSM medium without any carbon source. Mobilization of TAG was further monitored over a 48 h-kinetic. Bacterial samples were recovered at various time points and their TAG content analysed by TLC (Fig. 3A). Both *M. smegmatis* and *M. abscessus* were able to degrade 74 ± 9% and 59 ± 5% of their stored TAG, respectively, within the first 24 h of incubation. TAG consumption started rapidly after the first hours of starvation. Interestingly, relative TAG content as a function of time for each species (Fig. 3A) showed a linear relationship, suggesting that TAG hydrolysis occurred at a constant rate in both species during the first 24 h of carbon starvation. Accordingly, *M. smegmatis* and *M. abscessus* were able to hydrolyse 3.1% and 2.5% of their respective TAG content per hour during carbon starvation (Fig. 3A).

TAG consumption also depends on the expression of lipases, leading to the release of free fatty acids (FFA), subsequently fuelling pathways supporting survival and/or bacterial proliferation^40,41^. Since LipY (Rv3097c) has been described as the major lipase involved in TAG acquisition from the host but also for ILI breakdown in *M. tb*, we postulated that overproduction of the full-length LipY protein would affect TAG accumulation over time^40–42^. However using a *lipY*-overexpressing strain, we only observed a low decrease in the TAG level as compared to the control strain. Moreover, at later time points, 16 h and 24 h, TAG consumption was similar in both strains, thus suggesting that additional endogenous TAG lipases are probably operating (Fig. S5).

### ILI consumption can be pharmacologically blocked by using serine-hydrolase inhibitors

Chemical inhibitors can interfere with lipid metabolism by impairing the activity of mycobacterial lipolytic enzymes^43–45^. Pharmacological lipid inhibitors, such as serine-hydrolase inhibitors, have been widely used to decipher the molecular aspects of TAG metabolism and their relationship with the different mycobacterial metabolic stages^19,28,46,47^. To further confirm that mycobacterial lipases are responsible for ILI mobilization in carbon-deprived medium, bacteria were either treated with tetrahydrolipstatin (THL) or the oxadiazolone compound M*m*PPOX, two well-defined inhibitors of mycobacterial lipolytic enzymes^28,43,45,46,48^. Lipid-rich *M. smegmatis* grown in MSM NL Gly 1% was re-suspended in MSM Gly 1% and pre-incubated with either 5 µg/mL THL or 15 µg/mL M*m*PPOX. After 4 h, cells were washed, re-suspended in MSM medium containing increasing concentration of inhibitors and further incubated for 24 h at 37 °C with shaking. Apolar lipids were extracted and TAG levels were analysed by TLC (Fig. 3B). Pre-incubation with each inhibitor or DMSO (control) failed to alter TAG accumulation within ILI after 4 h (Fig. 3B). After 24 h in MSM, untreated cells or DMSO-treated cells showed a pronounced consumption of their TAG content of 75 ± 6% and 82 ± 4%, respectively. Conversely, when bacteria were incubated with THL, the TAG levels ranged from 0.8 ± 0.1 (5 µg/mL THL) to 1 ± 0.1 (50–100 µg/mL THL), suggesting that abrogation of the lipolytic activity has occurred (Fig. 3B). Similarly, following 24 h incubation with M*m*PPOX, TAG levels were 0.6 ± 0.05 for the lowest dose (15 µg/mL) and 0.8 ± 0.1 for the highest dose (150–300 µg/mL) (Fig. 3C). These findings suggest that, besides being a potent inhibitor of TAG lipolysis, M*m*PPOX appears less efficient than THL at blocking TAG consumption.

### Nitrogen-deprived and lipid-loaded M. abscessus are phenotypically tolerant to cefoxitin but not amikacin or clarithromycin

To investigate whether nitrogen starvation and carbon excess modulate drug tolerance in *M. abscessus*, antibiotic susceptibility testing was assessed using standard drugs used to treat *M. abscessus* infection. The drug susceptibility profile to amikacin (AMK), clarithromycin (CLR) and cefoxitin (FOX) was determined using the *M. abscessus* WT CIP104536^T^ (S) strain, the Δ*tgs1* mutant and its complemented counterpart grown either in Middlebrook 7H9 or in MSM NL Gly 1%. Incubation in a nitrogen-deprived medium did not affect the antibacterial activity of CLR and AMK, two drugs known to inhibit protein synthesis (Table 2). However, in MSM NL Gly 1% medium, *M. abscessus* became more tolerant to the β-lactam antibiotic FOX, with an approximate 2 to 4-fold increase in the MIC_90_ value (Table 2). Surprisingly under these experimental conditions, deletion of the *tgs1* gene did not change the susceptibility of the strain to FOX, suggesting that the observed antibiotic tolerance may be mediated by other mechanisms that are unrelated to TAG accumulation. Alternatively, one cannot exclude that the remaining pool of TAG characterizing Δ*tgs1* after 24 h (Fig. 2D) could be sufficient to trigger a decrease in the MIC_90_ and further work is required to validate this proposed mechanism.

**Table 2:** Drug susceptibility testing of lipid-poor (7H9^OADC^) and lipid-rich (MSM NL Gly 1%) *M. abscessus* cells.

*Culture conditions*	MIC_90_ (µg/mL)			
		AMK	FOX	CLR
7H9^OADC^ – 24 h	*M. abscessus* S	8.4	9.0	4.0
	*M. abscessus* S Δ*tgs1*	17.7	14.5	11.3
	*M. abscessus* S Δ*tgs1*-Comp	9.5	8.9	3.6
MSM NL Gly 1% – 24 h	*M. abscessus* S	3.7	>30	1.4
	*M. abscessus* S Δ*tgs1*	4.2	>30	1.1
	*M. abscessus* S Δ*tgs1*-Comp	4.7	>30	1.3

^a^MIC_90_: minimal compound concentration leading to 90% bacterial growth inhibition determined based on the REMA assay. Values are mean of two experiments performed in duplicate (CV% < 5%).

### Nitrogen limitation induces hypervirulence of M. abscessus in zebrafish embryos

**Figure 4:**
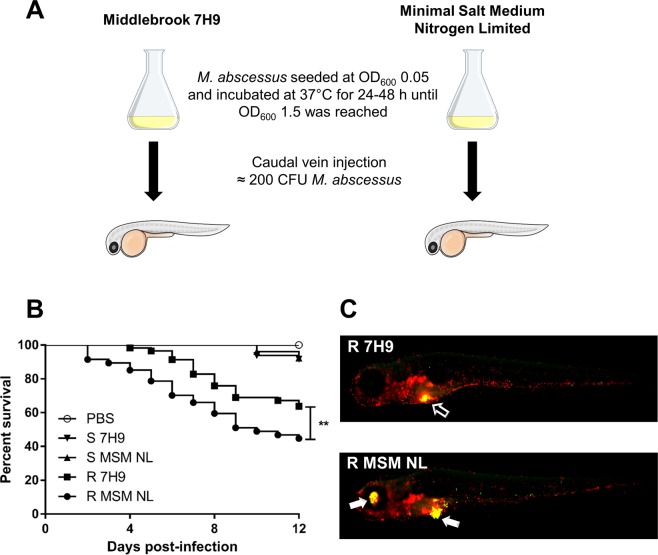
Nitrogen limitation induces hypervirulence in *M. abscessus*. **(A)** Simplified diagram of the experimental workflow used to induce lipid-loaded *M. abscessus* by nitrogen deprivation prior to infection of zebrafish embryos. Zebrafish embryos were infected *via* caudal vein injection at 30 hpf with approximately 200 CFU of *M. abscessus* S or R cultured for 48 h in either 7H9 or MSM NL Gly 1%. **(B)** Nitrogen limitation results in significantly earlier mortality in *M. abscessus* R-infected zebrafish. Zebrafish embryo survival was monitored daily over a 12-day period following infection. Each group consisted of approximately 20 embryos, with each curve reproduced in triplicate. Statistical analysis was completed using the Mantel-Cox log-rank test. **(C)** Representative images displaying significantly increased pathology phenotypes at 6 dpi in zebrafish embryos infected with the *M. abscessus* R morphotype following nitrogen limitation. Transgenic reporter line zebrafish embryos harbouring fluorescent macrophages (*mpeg:mCherry*) (red overlay) were infected with *M. abscessus* harbouring pTEC15::*mWasabi* (green overlay) and the merge was observed in yellow. The open arrow (top) displays an intact abscess, while the closed arrow (bottom) displays a ruptured abscess. Scale bars represent 500 µm. **p-value ≤ 0.01.

The granuloma represents a nutrient-deprived and oxygen poor microenvironment designed to restrict mycobacterial growth^49^. Consequently, pathogenic mycobacteria have developed unique survival strategies to sequester carbon-based energy sources to persist for prolonged periods within these harsh growth conditions^7^. Although it is hypothesized that TAG accumulation and ILI formation may be advantageous to long-term persistence, it is unclear whether this may confer a significant advantage in the establishment and outcomes of infection. To determine if nitrogen limitation is intrinsically linked to *M. abscessus* virulence, we thus examined if zebrafish embryo survival was altered following infection with *M. abscessus* R or S variants grown under nitrogen limited conditions (Fig. 4A). Prior performing infection experiments, we assessed that both R and S morphotypes were able to accumulate large amount of TAG when grown under these specific conditions (Fig. S4). When the R variant was grown in MSM NL Gly 1%, earlier death and significantly greater embryonic mortality was observed compared to the corresponding R strain cultured in 7H9^OADC^ medium (*p*-value* < *0.01) (Fig. 4B). Moreover, markedly increased disease symptoms were observed at 6 days post-infection (dpi) with the R variant subjected to nitrogen deprivation *vs*. standard culture conditions in 7H9^OADC^ (Fig. 4C). In contrast, there were no discernible differences in the embryonic mortality following *M. abscessus* S infection, irrespective of culture conditions (Fig. 4B).

### Nitrogen limitation leads to increased bacterial burden and enhanced granuloma formation

**Figure 5:**
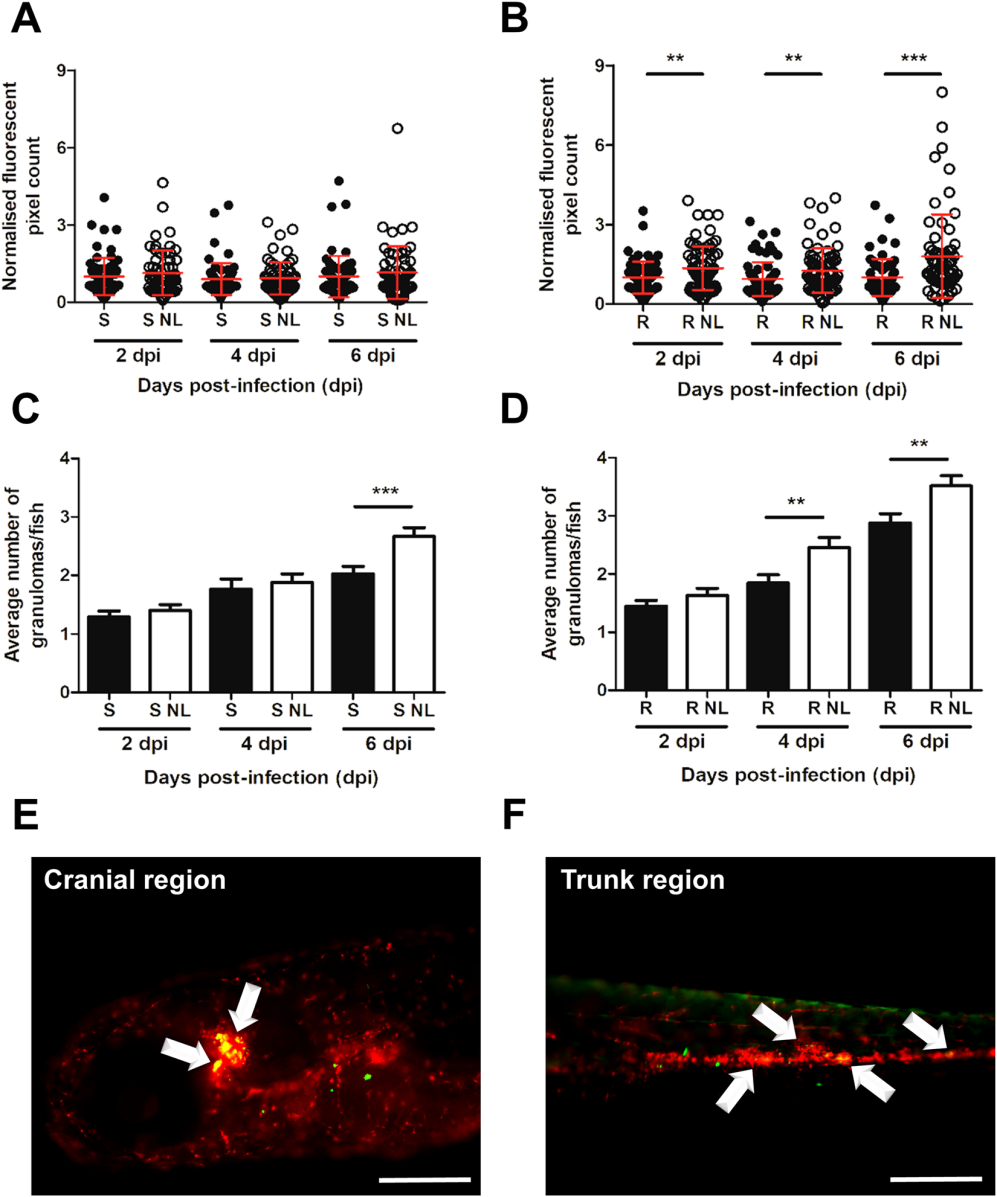
Nitrogen limitation results in increased bacterial burden and granuloma abundance. Transgenic zebrafish embryos harbouring red fluorescent macrophages (*mpeg1:mCherry*) were infected with approximately 200 CFU of *M. abscessus* S or R (producing mWasabi fluorescent protein) cultured in either 7H9 or in MSM NL Gly 1% and were imaged at 2, 4 and 6 dpi to quantify bacterial burden and granuloma number. **(A**,**B)** Bacterial burden was quantified using ImageJ ‘Analyse Particles’ function to determine fluorescent pixel counts. Error bars represent standard deviation, with each data point representing a single embryo. Data shown represent a pool of three individual experiments with approximately 20 embryos per group. Statistical analysis was completed using a Student’s t-test. **(C**,**D)** Granuloma number was quantified manually following colocalization (yellow) of bacteria (green) and macrophage aggregates (red) using ImageJ. Error bars represent standard deviation. Data shown is the average of three individual experiments with approximately 20 embryos per group. Statistical analysis was done using a Student’s t-test. **(E**,**F)** Representative images of granuloma quantification following bacterial and macrophage colocalization in the cranial and trunk region at 6 dpi. White arrows highlight granuloma presence. Scale bars represent 200 µm. **p-value ≤ 0.01, ***p-value ≤ 0.001.

It has been demonstrated that culture-induced nutrient starvation of *M. tb* resulted in significant elevations of bacterial burden and associated lung pathology in infected mice^50^. To determine whether this may be a conserved feature of pathogenic mycobacterial species, and whether this may contribute to increased embryonic mortality, we next quantified the bacterial burden and granuloma abundance during the early stages of zebrafish infection. As expected, zebrafish infected with *M. abscessus* R morphotype following nitrogen limitation exhibited a substantial rise in bacterial burden at all time points up to 6 dpi (*p*-value* < *0.005) (Fig. 5B). In addition, a significant increase in the granuloma abundance of 1.3- and 1.2-fold at 4 and 6 days following infection with nitrogen limited R bacilli, respectively, as compared to the R variant cultivated in 7H9^OADC^ was observed (*p*-value* < *0.006) (Fig. 5D). Granulomas containing bacteria were observed in the cranial region (Fig. 5E) or in the trunk region (Fig. 5F). Interestingly, although nitrogen limitation in *M. abscessus* S morphotype did not result in changes to bacterial burden at any of the time points examined (Fig. 5A), a marked increase (1.3-fold) in the number of granulomas at 6 days post-infection was observed compared to the S bacilli grown in 7H9^OADC^ (*p*-value* < *0.001) (Fig. 5C).

## Discussion

In this study, using two mycobacterial species with distinct lifestyles, we demonstrated that simple modulation of nutrient availability rapidly triggers the formation/consumption of ILI. By using a well-defined minimal salt medium and modulation of the carbon/nitrogen ratio, we demonstrated that nitrogen limiting conditions drastically impacted lipid metabolism in mycobacteria. More particularly, a high C/N ratio enhanced the intracellular TAG pool by 3–4.5 times. As previously reported in several mycolic-acid producing *Actinobacteria* such as *Rhodoccocus* species^51,52^, we show hereby that the *in vitro* modulation of carbon and nitrogen levels influences TAG accumulation under the form of highly distinguishable cytoplasmic structures in *M. smegmatis* and *M. abscessus*. Thanks to TEM, it was possible to identify a population of bacteria that were highly elongated and harboured several large ILI (Figs 1F and 2C). Similar phenotypes were reported within *M. tb* or *M. avium* infected-FMs, where bacteria accumulating large amounts of ILI rapidly stopped dividing but kept elongating^8,13^. Accordingly, this simple model of TAG accumulation triggered under nitrogen limiting conditions is particularly attractive, representing a powerful tool to dissect the physiological role of TAG accumulation/consumption during mycobacterial infection.

ILI formation in *M. tb* has been described as an adaptation strategy promoting survival during periods of non-replicating persistence *in vitro* but also *in vivo*, predominantly mediated by the “dormancy” regulon^32,53–55^ and directly related to the overproduction of the TAG synthase 1 (Tgs1)^17,31,56,57^. By using our nitrogen limiting model, we confirmed that the *M. abscessus* Δ*tgs1* deletion mutant^26^ failed to accumulate TAG, in agreement with previous work in FM^26^ (Fig. 2D). This result not only highlights a major role of this enzyme in ILI formation, but strongly supports our hypothesis that our “*in vitro*” model closely mimics the unique host-pathogen interactions occurring during FM infection.

Because TAG hydrolysis within ILI is required for reactivation of *M. tb* replication by rapidly generating energetic metabolites, we addressed whether ILI degradation was also a reversible phenomenon in this system. Following ILI formation, incubation of bacteria in carbon-starved conditions and treatment with serine-hydrolase inhibitors, THL^19,23,28,46,58^ or M*m*PPOX^43,48^, resulted in nearly complete blockage of TAG lipolysis, even at low concentrations. This supports the fact that lipid hydrolysis is mediated by specific mycobacterial TAG-lipases. Until now, only two intracellular lipases have been identified as being involved in ILI degradation: BCG1721, an ILI-associated bi-functional enzyme from *M. bovis* BCG exhibiting both lipase and acyl-CoA synthase (ACSL) activities^20^ and the well-studied *M. tb* triacylglycerol hydrolase LipY (Rv3097c), recognized as a major enzyme for TAG hydrolysis within ILI^40–42,57,59^. In agreement with previous work^40^, we confirm here that in a *M. smegmatis* strain constitutively overexpressing the *M. tb* LipY protein, increased TAG hydrolysis occurs. That after 24 h of carbon starvation there were no significant differences between strains harbouring pVV16::*lipY* or the empty vector, may suggest that additional endogenous lipases are involved in this process. Notably, no *lipY* orthologues are found within the *M. smegmatis* and *M. abscessus* genomes. Conversely, MSMEG_3767 and MAB_2348 share 71.5% and 65.1% amino-acid sequence identities with BCG1721, respectively. Thus, orthologues of BCG1721 and/or additional uncharacterized mycobacterial lipases may degrade TAG contained within ILI.

Defining key physiological mechanisms underlying bacterial persistence during active TB treatment, relapse or failure is crucial. It is now well-established that slow or non-replicating bacteria with reduced metabolic activity, qualified as “dormant” are more recalcitrant to antibiotic treatments^7,10,17^. Similarly, cultures in late stationary phase are more tolerant to antibiotics than exponential growing cells^11,60,61^. Understanding the potential relationship between phenotypic antibiotic tolerance and the presence of ILI within mycobacteria is an intriguing challenge. Several studies have emphasized that TAG accumulation under the form of ILI drastically increase tolerance to first-line TB drug*s*^11,17,57^. In our nitrogen-deprivation model, while the intracellular level of TAG in *M. smegmatis* can be directly related to the antibiotic susceptibilities (Table 1), it was difficult to establish a clear relationship between lipid-rich phenotype and antibiotic tolerance using *M. abscessus* (Table 2).

Zebrafish embryos infected by the *M. abscessus* R variant harbouring a lipid-rich phenotype exhibited significantly increased mortality, thus providing evidence, for the first time, that the presence of ILI may prime mycobacteria in their ability to establish infection within the host. Intriguingly, although this was not the case for the S morphotype, known to be primarily avirulent in the zebrafish embryo infection model, granuloma abundance was significantly increased with lipid-rich bacteria, regardless of the morphotype^37^. In addition, bacterial burden was significantly increased in lipid-loaded R morphotypes at all time points examined, however this was not conserved in S morphotype, suggesting that lipid utilization pathways may differ between morphotypes. Finally as observed for *M. tb*, it cannot be excluded that lipid rich *M. abscessus* could be found within patients sputum and that this phenotype may potentially affect the human-to-human transmission process through aerosolization^9,62^.

In conclusion, we have confirmed that nitrogen deprivation promotes TAG accumulation in mycobacteria, resulting in the production of lipid-loaded cells that appear phenotypically similar to those found within foamy macrophages. Overall, our results strongly support the view that ILI in mycobacteria act as specialized and highly conserved structures dedicated to provide a rich energy and carbon source *in vivo*. Importantly, being reversible, this model provides new perspectives to define the biological functions of ILI during the mycobacterial lifecycle. Coupling either TAG accumulation/consumption with RNAseq or proteomics analyses would allow to add new light into the general physiology of mycobacteria as they become ILI-enriched or as they utilize available TAG pools, particularly suited for the discovery and characterization of new lipolytic enzymes participating in these processes.

## Material and Methods

### Bacterial strains and classical media

*Escherichia coli* DH5α was cultured in Lysogeny broth or onto solid medium (Invitrogen, France). For classical culture conditions, *M. smegmatis* mc^2^155^63^ was grown in Middlebrook 7H9 complete medium containing 0.05% Tween-80 and 0.2% Gly (7H9). *M. abscessus* CIP104536^T^, S and R morphotypes, were cultured in 7H9 medium containing 10% BBL™ Middlebrook OADC Enrichment (7H9^OADC^). When required, kanamycin and hygromycin B (Euromedex, France) were added to the medium at a final concentration of 100 µg/mL for mycobacteria and 50 µg/mL and 200 µg/mL, respectively, for *E. coli*. Bacterial strains used in this study are listed in Table S1.

### Generation of lipid-loaded cells

Mycobacterial species were pre-cultured in their respective classical media at 37 °C and 200 rpm as described above. Then fresh medium was inoculated to an initial OD_600nm_ of 0.05 and further incubated at 37 °C and 200 rpm until the OD_600nm_ reached 1–1.5. Bacterial cells from exponential phase were harvested for 10–15 min at 5,000 *g*. Pellets were washed once with sterile Phosphate Buffer Saline (PBS) buffer pH 7.4 containing 0.05% Tween-20 (PBS-T), once with classic PBS buffer and finally normalized and re-suspended at OD_600nm_ = 10 in PBS. This solution of bacteria-containing PBS was used to inoculate at an initial OD_600nm_ of 0.05–0.1 either fresh 7H9 medium, Minimal Mineral Salt Medium (MSM) (2 g/L Na_2_HPO_4_, 1 g/L KH_2_PO_4_, 0.5 g/L NaCl, 0.2 g/L MgSO_4_, 20 mg/L CaCl_2_ and 1 g/L NH_4_Cl) or Mineral Salt Medium Nitrogen Limiting (MSM NL) (containing only 0.05 g/L NH_4_Cl). In experiments where the effect of carbon source concentration was studied, each medium was supplemented with 1, 2 or 5% (Gly) (*v*/*v*). To avoid bacterial clumping in MSM, Tyloxapol (Sigma) was added at a final concentration of 0.02% (*v*/*v*).

### Normalization, lipid extraction and TLC analysis

*M. smegmatis* and *M. abscessus* cells were grown in defined culture medium as described above with specific carbon sources and concentrations. At specific time points, cultures were centrifuged at 4,000 *g* for 10 min at 4 °C. Cells were washed three times in distilled water, heated at 80 °C for 30 min and removed from the BSL-2 laboratory. Pellets were further lyophilized overnight and weighed to obtain the exact dry weight of the mycobacterial residue. Apolar lipids were extracted as previously described^64^. Briefly, 2 mL of MeOH-0.3% NaCl (10:1, *v/v*) was added per 50 mg dry extract. The saline-MeOH solution containing the bacterial dry extract was mixed for at least 15 min with 1 mL petroleum ether in Pyrex^®^ tubes at room temperature (RT) using a tube rotator. After centrifugation at 3,000 *g* for 5 min, the upper organic layer was transferred to a fresh tube. This step was repeated three times and a final centrifugation was completed for 15 min at 3,000 *g* to remove residues carried over during the extraction. The upper organic layer containing apolar lipids was transferred to a fresh pre-weighed vial, and the solvent was evaporated to dryness under a stream of nitrogen. Finally, the obtained dry apolar lipid residue was re-suspended in 300 µL dichloromethane.

Thin layer chromatography using aluminium TLC plates (Silica Gel 60, Merck) allowed analysis of the obtained extracted lipids. The solvent mixture used in the TLC analysis of TAG was petroleum ether (40–60 °C fraction)/diethyl ether (90:10 *v*/*v*). The spots were visualized by dipping the plate into a solution of 10% phosphomolybdic acid in absolute ethanol followed by heating at 120 °C in an oven for 5–10 min. Each resolved plate was scanned using a Chemidoc^TM^ MP Imaging System (Bio-Rad), and densitometric analyses were performed using the ImageLab^TM^ software version 5.0 (Bio-Rad) to determine relative TAG content per sample. In graphs, histograms represented the mean ± standard deviation of at least two or three independent experiments. Differences were considered statistically significant when *p*-values tested ≤ 0.05 with a one-way ANOVA.

### Nile Red staining and fluorescence microscopy

Mycobacterial cultures were assessed for ILI formation using Nile Red staining as previously described^28^ with slight modifications. Approximately 7.5 × 10^7^ mycobacterial CFU were centrifuged in Eppendorf tubes for 5 min at 5,000 *g*. Pellets were washed twice with 500 µL PBS buffer and re-suspended in 300 µL of PBS. After, 15 µL (0.5 mg/mL in absolute ethanol) of Nile Red fluorescent dye (Interchim) was added to the cell suspension. Cells were further incubated for 20-30 min at 37 °C in the dark. Stained bacteria were harvested for 5 min at 5,000 *g*, then washed twice with 500 µL of PBS-0.05% Tween 80 buffer and finally re-suspended in 300 µL of PBS. Bacterial suspensions (5 µL) were spotted between a coverslip of 170 µm thickness and a 1.5% agarose-PBS pad. Bacteria were analysed by snapshot imaging at room temperature using an Olympus FV1000 confocal microscope coupled with ×100 oil-objective. Exposure time was 800 ms for both phase-contrast and fluorescence images (λ_exc_\λ_em_ = 540/620 ± 10 nm) with conserved settings (X, Y, Z, Gain and PFS-offset). Images recorded (126.98 µm × 126.98 µm; 512 × 512 pixels) were processed using the open source program ImageJ 1.51 K (NIH, USA). Analysis of average fluorescence intensity was calculated using ImageJ 1.51 K software with five snapshots from two independent cultures stained on independent occasions. GraphPad Prism 4 was used for performing statistical analyses, where differences in fluorescence intensity were considered significant when the calculated *P*-values were smaller than 0.05 using a two-tailed Mann-Whitney test.

### Processing for electronic microscopy

Bacteria were fixed at room temperature with 2.5% glutaraldehyde (Sigma) in Na-cacodylate buffer 0.1 M (pH 7.2) containing 0.1 M sucrose, 5 mM CaCl_2_, and 5 mM MgCl_2_, washed with complete cacodylate buffer and post-fixed for 16 h at room temperature with 1% osmium tetroxide in the same buffer without sucrose^65^. Finally, samples were washed twice in cacodylate buffer and dehydrated in a graded series of ethanol solutions and gradually incorporated in Spurr resin. Thin sections (80 nm thick) were stained with 1% uranyl acetate in distilled water and then with lead citrate before being observed by electron microscopy. Image acquisition was performed with a FEI Tecnai G2 20 TWIN 200 kV Transmission Electron Microscope, using a Lab6 cathode and an Eagle 2k camera. Images were processed using the open source program ImageJ 1.51 K (NIH, USA). Concerning statistical analysis, between 300 and 400 mycobacteria per sample were examined under a TEM to determine the percentage of each category of *M. smegmatis* or *M. abscessus* ILI profiles. Cells were examined at random, and care was taken to avoid serial sections. Mycobacteria were previously divided into 3 different categories, according to the number/size of ILI^8,26^. Bacteria displaying no or few small ILI (0.1 µm in width) were classified as ILI^0/1+^ profiles; bacteria containing few ILI with medium size (0.2–0.3 µm in width) were classified as ILI^2+^; and finally, bacteria where almost all the cytoplasm is occupied by large ILI (0.3–0.5 µm in width) were classified as ILI^3+^ (Fig. S3).

### Carbon starvation and ILI hydrolysis

For studies concerning lipid mobilization, mycobacterial species were cultivated in MSM NL containing Gly as carbon source for 48 h. These lipid-rich bacteria were centrifuged for 10 min at 5,000 *g*, washed twice in PBS buffer and finally re-suspended in MSM medium without any carbon source in order to trigger starvation. Cells were then incubated at 37 °C, with shaking at 200 rpm and finally collected at specific time points and treated as described above lipid analysis by TLC. For inhibition studies, bacteria were pre-incubated with THL or M*m*PPOX in MSM Gly medium for 4 h, centrifuged for 10 min at 4,000 *g* and finally re-suspended in MSM medium devoid of carbon for 24 h with the appropriate concentration of THL (5 mg/mL stock solution in DMSO) or M*m*PPOX (5 mg/mL stock solution in DMSO). Cells were collected at specific time points in order to analyse their lipid content.

### *lipY* overexpression and *tgs* mutants and complemented strains

*M. abscessus S Δtgs1*, *Δtgs2* mutants and their respective complemented counterparts used in this study (Table 1) were generated previously by using the recombineering method of allelic exchange^26^. The ORF encoding the LipY protein (gene *Rv3097c*) was amplified by PCR using Phusion Hot Start Polymerase (Thermo-Scientific), *M. tb* H_37_Rv genomic DNA and specific primers (Forward 5′ ggaatcacttcgcatatggtgtcttatgttgttgcgttgcc 3′ and Reverse 5′ gtggtggtggtgaagcttggcggcgataccgagttgctg 3′, with incorporated NdeI and HindIII restriction sites at the 5′ends, respectively). PCR fragments were digested, purified and cloned into the pVV16 mycobacterial expression vector to create pVV16::*lipY*. The recombinant plasmid was checked by sequencing (GATC, Biotech) and 400 ng of pVV16 and pVV16::*lipY* were used to transform electrocompetent *M. smegmatis* cells prepared as previously described^66^. Recombinant clones were selected on 7H9 Middlebrook agar medium supplemented with hygromycin and kanamycin. Protein production was confirmed by immunoblotting. Briefly, total lysates were loaded and separated on a 12% SDS-PAGE gel and transferred onto a nitrocellulose membrane using a Trans-Blot Turbo Transfer System (Bio-Rad). Immunoblotting of _6_His-tagged proteins was performed using the HisProbe^TM^ HRP conjugate (Thermo-Scientific). MSMEG_0220 was used as a housekeeping protein control that was immunodetected with rabbit polyclonal antibodies directed against the *M. tb* Rv0183 protein^67,68^ and horseradish peroxidase-conjugated anti-rabbit IgG (Thermo-Scientific). Revelation was done with the Pierce^TM^ ECL Western Blotting substrate solution (Thermo-Scientific) and Western Blots were visualized with a ChemiDoc^TM^ MP Imaging System (Bio-Rad).

### Drug susceptibility testing

Susceptibility testing was performed using the Middlebrook 7H9 broth microdilution method with slight modifications. All assays were carried out at least in duplicate. MICs were determined in 96-well flat-bottom Nunclon Delta Surface microplates with lids (Thermo-Fisher Scientific, ref. 167008) using the resazurin microtiter assay (REMA)^69,70^. Briefly, log-phase *M. smegmatis* bacteria were diluted to a cell density of 5 × 10^6^ cells/mL in classic 7H9, in MSM Gly 1% or MSM NL Gly 1% medium. Then, 100 µL of the above inoculum was added to each well containing 100 µL each respective medium, serial two-fold dilutions (from 30 µg/mL to 0.04 µg/mL) of the selected inhibitor, kanamycin (KAN), isoniazid (INH), rifampicin (RIF), amikacin (AMK), cefoxitin (FOX), clarithromycin (CLR) or controls to a final volume of 200 µL (final bacterial charge of 2.5 × 10^6^ cells/mL per well). Growth controls containing no inhibitor (*i.e*., bacteria only = B), inhibition controls containing 50 µg/mL KAN and sterility controls (*i.e*., medium only = M) without inoculation were also included. Plates were incubated at 37 °C in a humidity chamber^71^ to prevent evaporation for 3–5 days. Then, 20 µL of a 0.025% (*w/v*) resazurin solution was added to each well, and the plates were incubated at 37 °C for colour change from blue to pink or purple and for a reading of fluorescence units (FU). Fluorescence corresponding to the resazurin reduction was quantified using a Tecan Spark 10 M multimode microplate reader (Tecan Group Ltd, France) with excitation at 530 nm and emission at 590 nm. For fluorometric MIC determinations, a background subtraction was performed on all wells with a mean of the M wells. Relative fluorescence units were defined as: RFU% = (test well FU/mean FU of B wells) × 100. MIC values were determined by fitting the RFU% sigmoidal dose-response curves in Kaleidagraph 4.2 software (Synergy Software)^70,72^. The lowest drug concentrations inhibiting 90% of growth was defined as the MIC_90_.

### Phenotypic antibiotic resistance by plate count method (CFU counting)

The phenotypic antibiotic resistance against different concentrations of RIF, INH and KAN was measured by the plate count method (CFU counting). A 24 h-logarithmic-phase *M. smegmatis* culture in either 7H9 or MSM NL Gly 1% medium was diluted to an OD of around 0.05 (*i.e*., 5 × 10^6^ cells/mL) and split into 5 mL samples, then drugs were added at either 10 µg/mL (KAN) (corresponding to 10× MIC) or 12 µg/mL (INH, RIF) (corresponding to 4 × MIC), with an untreated sample serving as a control. Each sample was re-incubated for an additional 24 h at 37 °C and 200 rpm. Serial dilutions of each culture were then plated on 7H9 agar medium. Colonies were counted after 4 to 5 days of incubation at 37 °C to check bacterial viability (adapted from^73^).

### Zebrafish breeding and ethics statement

Zebrafish experiments were completed in accordance with the guidelines defined by the European Union for the use of laboratory animals. All animal experimentation was approved by the Direction Sanitaire et Vétérinaire de l’Hérault et Comité d’Ethique pour l’Expérimentation Animale de la région Languedoc Roussillon under the reference CEEA-LR-1145. Adult zebrafish were housed at the Centre National de la Recherche Scientifique, Montpellier, France. Zebrafish embryos were obtained by natural spawning and maintained at 30 °C in 60 µg/mL ocean salts. Zebrafish experiments were performed using the *golden* mutant line^74^ or transgenic tg(*mpeg1:mCherry*) line^37,75^.

### Zebrafish microinjection

Zebrafish infection experiments were performed as previously described with minor modifications^37^. At approximately 24 h post-fertilisation (hpf), embryos were dechorionated following 3 min incubation with 1 mg/mL Pronase (Sigma-Aldrich). At 30 hpf, embryos were anaesthetized with 160 µg/mL tricaine (Sigma-Aldrich) prior to caudal vein injection with approximately 200 CFU *M. abscessus* S or R variants (CIP104536^T^) containing pTEC15 expressing the *mWasabi* gene under the control of a strong mycobacterial promoter (Addgene Plasmid #30174). A control cohort following a mock injection of sterile PBS was included in each experiment. Following microinjection, embryos were recovered in a separate petri dish at 30 °C with embryo mortality observed for a period of up to 12 days post-infection (dpi).

### Bacterial burden and granuloma quantification

For microscopy experiments, zebrafish embryos were anaesthetized in a tricaine solution and mounted in 3% methylcellulose (Sigma-Aldrich) for imaging on a Zeiss Axio Zoom.V16 coupled with an Axiocam 503 mono (Zeiss). Image acquisition and processing was completed using ZEN 2 software (blue edition). Bacterial burden was measured as the number of pixels contained within each embryo above the background fluorescence with the ImageJ software version 1.51w (National Institutes of Health). Fluorescent pixels were counted using the ‘Analyse particles’ function with threshold settings kept consistent for the duration of each experiment. To enumerate granuloma abundance, bacterial (green) and macrophage (red) fluorescent signals were merged using ImageJ to identify infection foci. Granulomas were counted manually and defined as an infection foci containing at least several macrophages aggregated at the site of bacterial infection. Graphpad Prism 5 was used to perform statistical analysis of all zebrafish experiments. For survival analysis experiments, Log-rank (Mantel-Cox) tests were applied. To examine bacterial burden and granuloma abundance, Student’s t-tests were applied.

## Supplementary information

Supplemental Data
