# Supplementary material for: Nitrogen deprivation induces triacylglycerol accumulation, drug tolerance and hypervirulence in mycobacteria
Source: Sci Rep. 2019 Jun 17;9:8667. doi: 10.1038/s41598-019-45164-5 (PMC6572852; doi:10.1038/s41598-019-45164-5)
Supplement: Supplementary file 1 — Supplemental Data [file 41598_2019_45164_MOESM1_ESM.docx]

**Supplementary Information**

**Nitrogen deprivation induces triacylglycerol accumulation, drug tolerance and hypervirulence in mycobacteria**

Pierre Santucci^1§^, Matt D. Johansen^2§^, Vanessa Point^1^, Isabelle Poncin^1^, Albertus Viljoen^2^, Jean-François Cavalier^1^, Laurent Kremer^2,3^ and Stéphane Canaan^1^*

^1^ Aix-Marseille Univ, CNRS, LISM, IMM FR3479, Marseille, France.

^2^ Institut de Recherche en Infectiologie de Montpellier (IRIM), CNRS, UMR 9004, Université de Montpellier, 34293 Montpellier, France

^3^ INSERM, IRIM, 34293 Montpellier, France

**Keywords**: Lipid metabolism, *Mycobacterium smegmatis*, *Mycobacterium abscessus*, lipase, TAG synthase, intracellular lipid inclusions, lipase inhibitor, persistence, zebrafish, granuloma.

**Running Title**: Nitrogen levels affect mycobacterial TAG synthesis

^§^ These authors have contributed equally to this work

* Corresponding author: email: [stephane.canaan@imm.cnrs.fr](mailto:stephane.canaan@imm.cnrs.fr), phone +33 4 91 16 40 93

| **Mycobacterial species** | **Strain** | **Morphotype** | **Source** |
| --- | --- | --- | --- |
| *Mycobacterium smegmatis* | mc^2^155 | - | Snapper et al. 1990*^a^* |
| *Mycobacterium abscessus sensu stricto* | CIP104536^T^ | Smooth (S) | Laboratoire de Référence des Mycobactéries (IP, France) |
| *Mycobacterium abscessus sensu stricto* | CIP104536^T^ | Rough (R) | Laboratoire de Référence des Mycobactéries (IP, France) |
| *Mycobacterium abscessus sensu stricto* | CIP104536^T^ pTEC15::*mWasabi* | Smooth (S) | This study |
| *Mycobacterium abscessus sensu stricto* | CIP104536^T^ pTEC15::*mWasabi* | Rough (R) | This study |
| *Mycobacterium abscessus sensu stricto* | CIP104536^T^ *Δtgs1* | Smooth (S) | Viljoen et al. 2016 *^b^* |
| *Mycobacterium abscessus sensu stricto* | CIP104536^T^ *Δtgs2* | Smooth (S) | Viljoen et al. 2016 *^b^* |
| *Mycobacterium abscessus sensu stricto* | CIP104536^T^ *Δtgs1::tgs1* | Smooth (S) | Viljoen et al. 2016 *^b^* |
| *Mycobacterium abscessus sensu stricto* | CIP104536^T^ *Δtgs2::tgs2* | Smooth (S) | Viljoen et al. 2016 *^b^* |

**Table S1: List of bacterial strains used in this study.**

*^a^* Snapper, S. B., Melton, R. E., Mustafa, S. , Kieser, T. and .Jacobs Jr W. R. Isolation and characterization of efficient plasmid transformation mutants of Mycobacterium smegmatis. *Mol Microbiol*, 4: 1911-1919. doi:10.1111/j.1365-2958.1990.tb02040.x (1990)

*^b^* Viljoen, A., Blaise, M., de Chastellier, C. & Kremer, L. MAB_3551c encodes the primary triacylglycerol synthase involved in lipid accumulation in Mycobacterium abscessus. *Mol Microbiol* **102**, 611-627. doi:10.1111/mmi.13482 (2016)


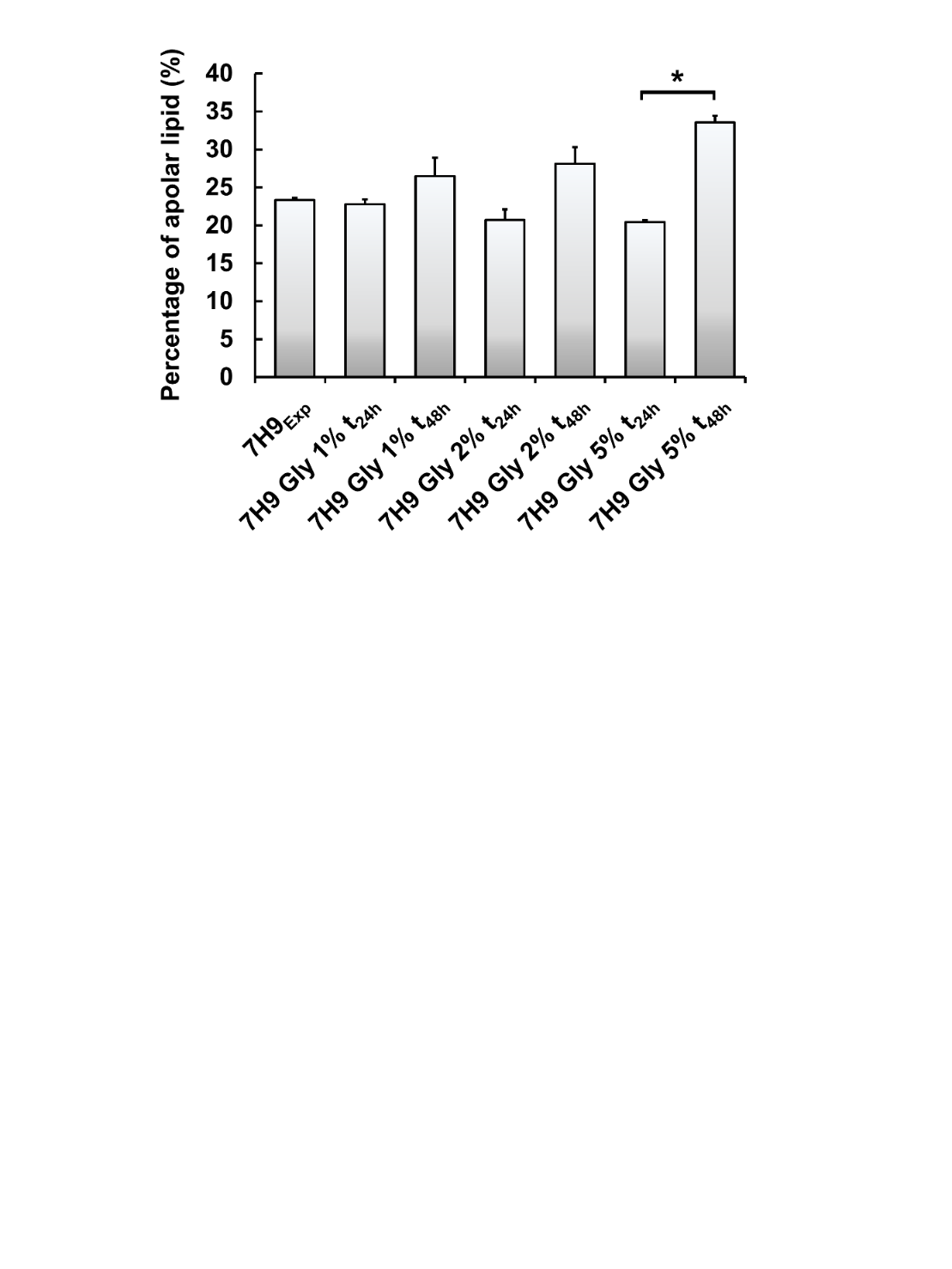


**Figure S1: Glycerol excess promotes apolar lipid synthesis in the stationary phase.** Mycobacterial cultures were grown in different media containing increasing glycerol (Gly) concentrations and were collected at 24 h or 48 h, corresponding to the exponential or stationary phase, lyophilized and equal amounts of dry cells used for subsequent apolar lipid extraction. After extraction, apolar lipids were weighed and expressed as % of total cell dry weight.


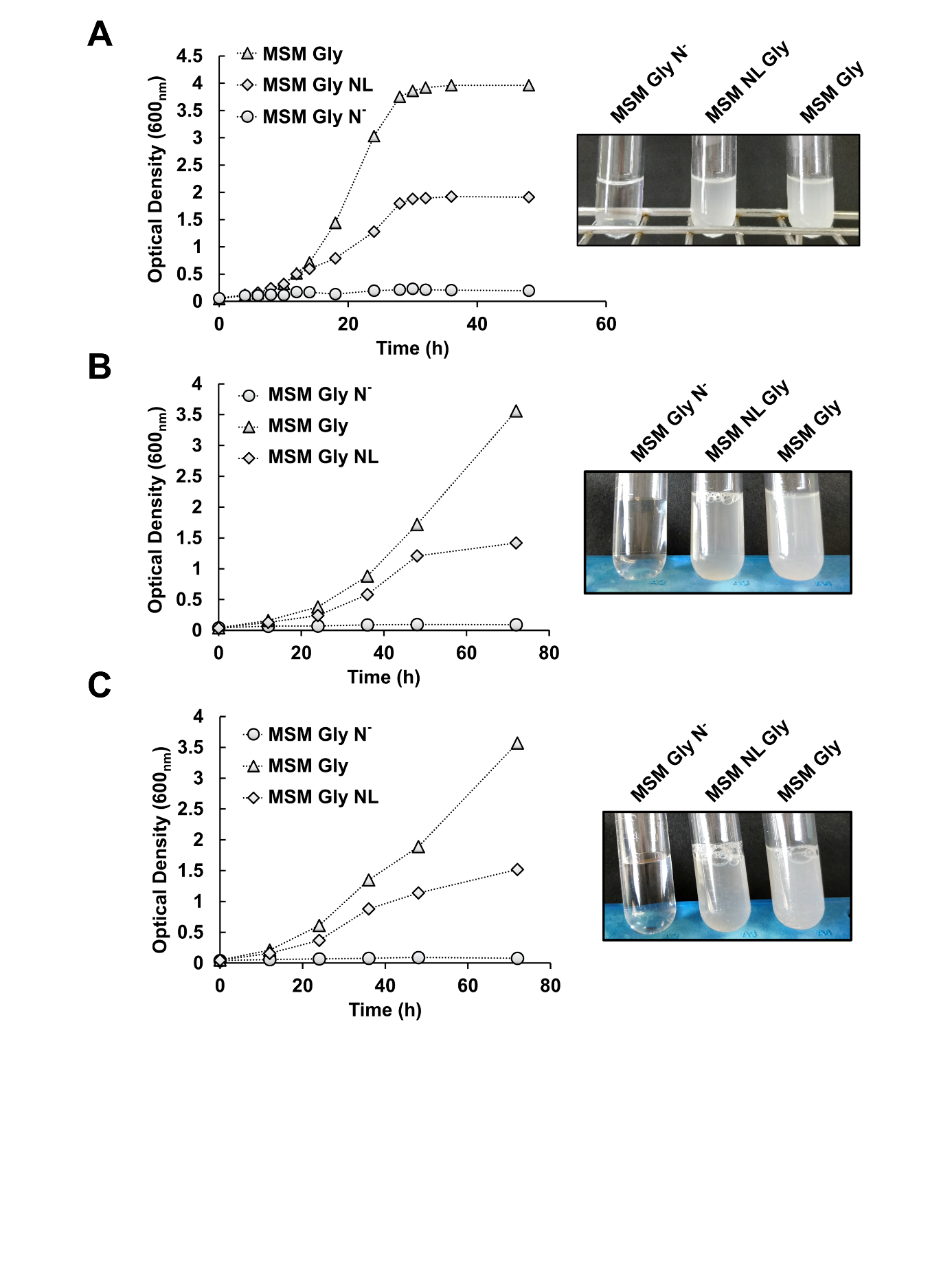


**Figure S2: Growth under a limiting nitrogen concentration (MSM NL Gly) drastically decreases the bacterial biomass at the stationary phase.** (**A**) *M. smegmatis* mc^2^155 (**B**) *M. abscessus*CIP104536^T^ (S morphotype) and (**C**) *M. abscessus* CIP104536^T^ (R morphotype) were grown in MSM containing either 1 g/L, 0.05 g/L or devoid of NH_4_Cl and supplemented with 1% of glycerol as carbon source (MSM Gly, MSM NL Gly, and MSM N^-^ Gly respectively). Media were inoculated at initial OD_600nm_ = 0.05, and growth was followed over a 48 h-72 h period. Pictures are demonstrating the impact of nitrogen limitation on mycobacterial growth at the end of the 48 h incubation period.


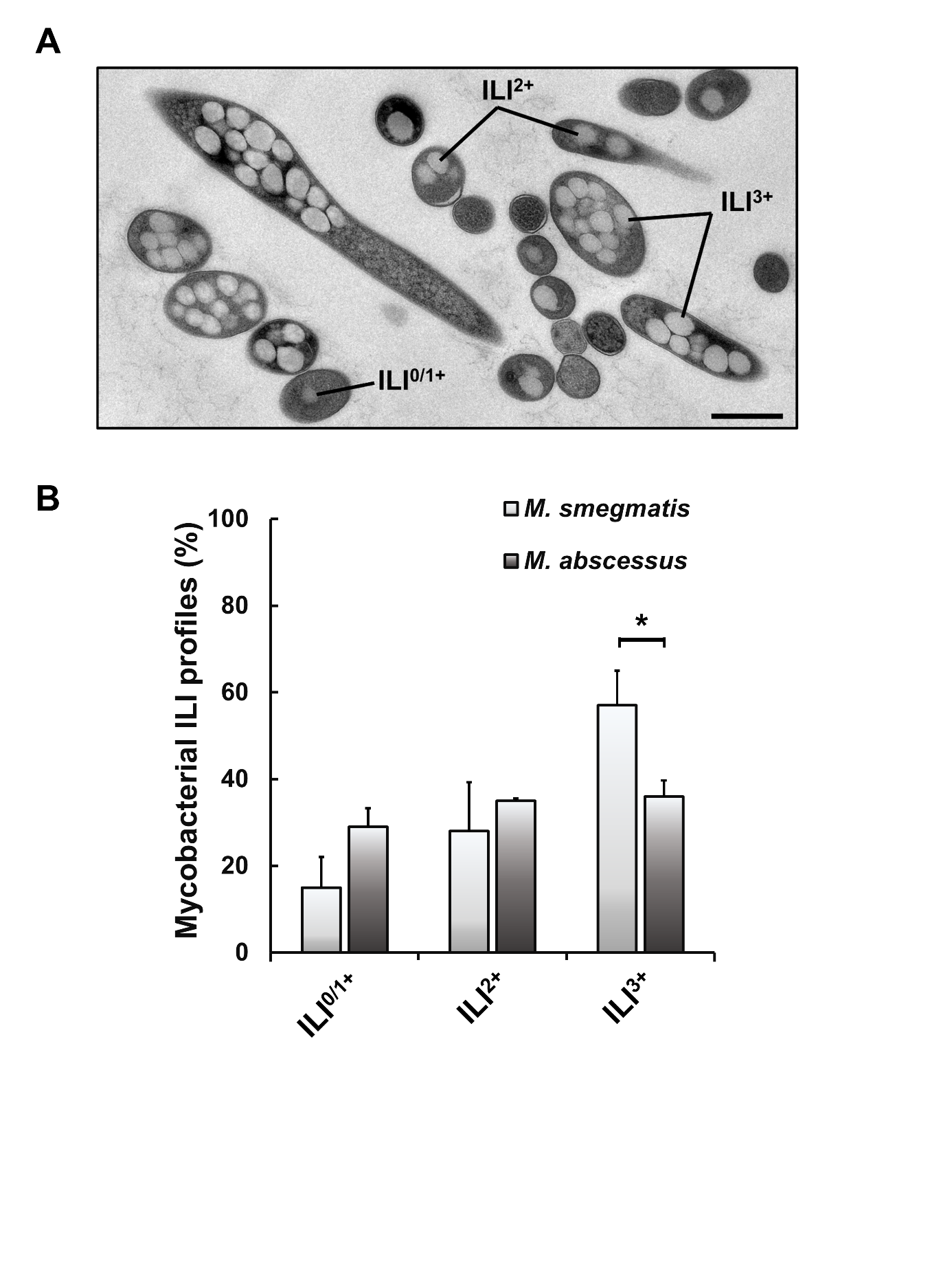


**Figure S3: Morphological features and distribution of ILI profiles among mycobacterial lipid-rich cells.** (**A**) *M. smegmatis* was grown in MSM containing 0.05 g/L of NH_4_Cl and supplemented by 1% of glycerol as carbon source for 24 h. Cells were fixed with glutaraldehyde and processed for EM. (**A**) Overview of mycobacterial ILI profiles in MSM NL Gly 1% medium, showing ILI^0/1+^; ILI^2+^ and ILI^3+^ ultrastructural features. Scale bar represents 2 µm. (**B**) Distribution of mycobacterial ILI profiles within *M. smegmatis* and *M. abscessus* populations. Results are expressed as mean values ± SD (in %) of distinct count from different EM grids (*n* = 400 cells).


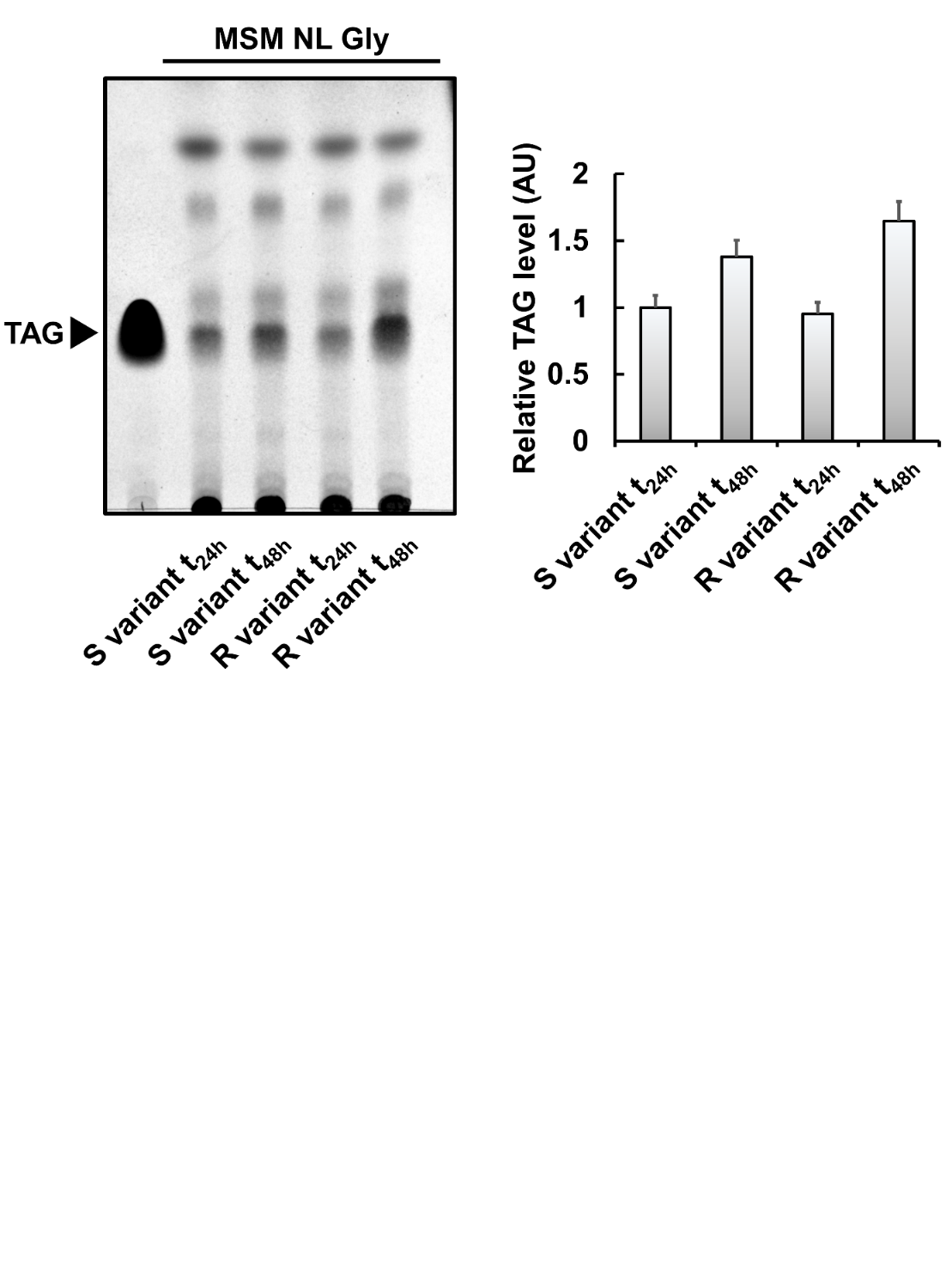


**Figure S4: Both *M. abscessus* S and R variants produce high amounts of TAG under nitrogen limitation.** *M.* *abscessus* S and R variants were grown in MSM containing 0.05 g/L of NH_4_Cl and supplemented with 1% of glycerol as carbon source for 48 h. Cultures were collected after a 24 h or 48 h incubation period, lyophilized and equal amounts of dry cells used for apolar lipid extraction. TAG levels from each culture were analysed by TLC with triolein as standard. TLC densitometric analysis was performed using the *M. abscessus* S variant at t_24h_ as reference. Results are expressed as mean values ± SD of two independent experiments.


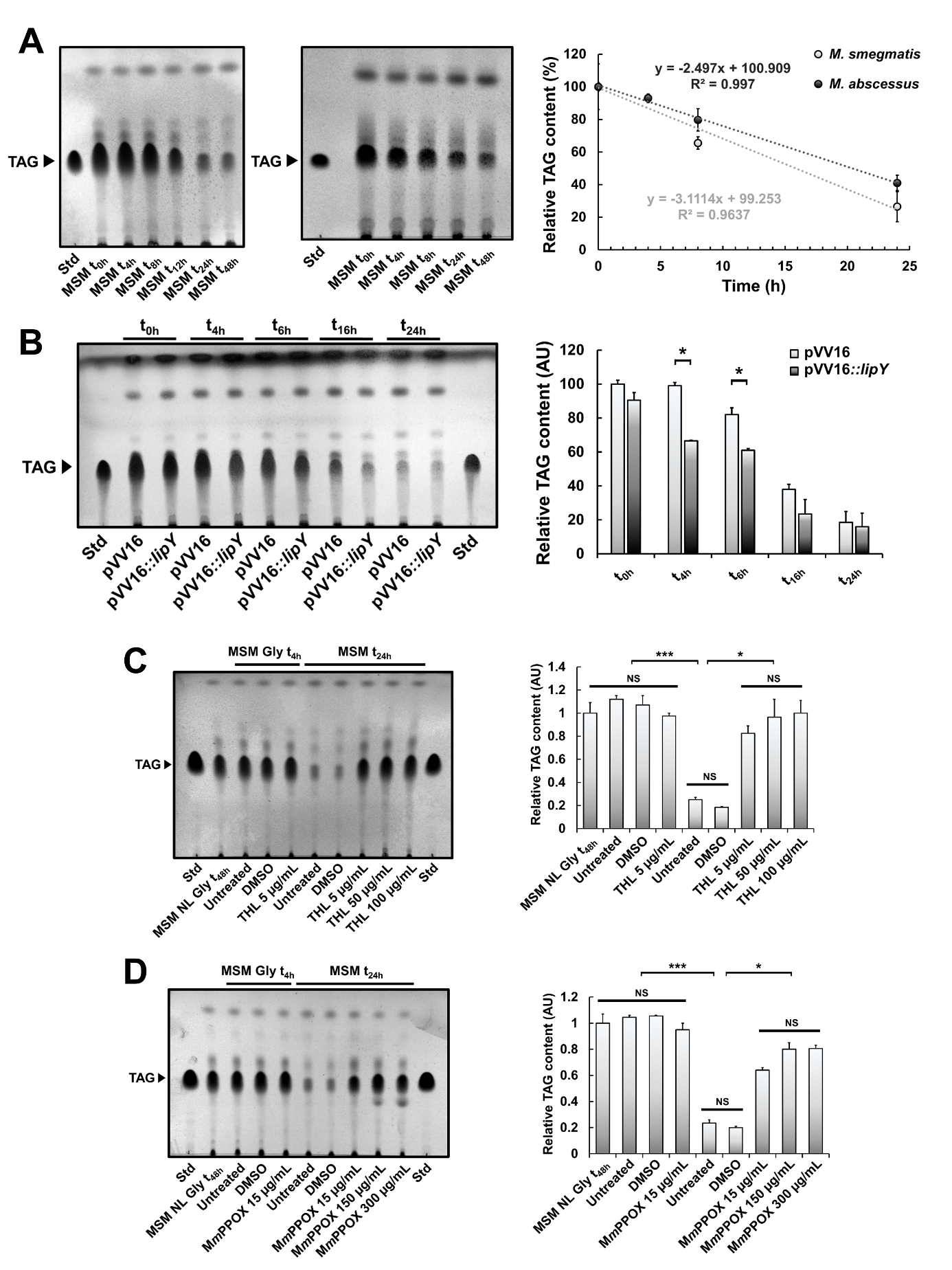


**Figure S5**: *M. smegmatis* strains harbouring either the empty pVV16 vector or pVV16::*lipY* were generated and grown for 48 h in MSM NL Gly 1% (to provide lipid-loaded bacilli) and re-suspended in MSM for monitoring the TAG profile at different time points (0, 4, 6, 16 and 24 h). TAG levels from each culture were analysed by TLC and TLC densitometric analysis was performed using the recombinant *M. smematis* strain containing the empty pVV16 vector as reference. Results are expressed as mean values ± SD of two independent experiments. TAG band intensity of pVV16 and pVV16::*lipY* strains were compared using a one-way ANOVA test where * corresponds to a *p*-value < 0.05.
